# Targeting p21-activated kinase 1 inhibits growth and metastasis via Raf1/MEK1/ERK signaling in esophageal squamous cell carcinoma cells

**DOI:** 10.1186/s12964-019-0343-5

**Published:** 2019-04-11

**Authors:** Liang Chen, Shuning Bi, Jiuzhou Hou, Zhijun Zhao, Chaojie Wang, Songqiang Xie

**Affiliations:** 1 Institute of Chemical Biology, College of Pharmacy, Henan University, Kaifeng, 475004 China; 2 Department of Medicine and Therapeutics, Luohe Medical College, Luohe, 462000 China; 3 The Key Laboratory of Natural Medicine and Immuno-Engineering, Henan University, Kaifeng, 475004 China

**Keywords:** ESCC, PAK1, Metastasis, IPA-3, Raf1, MEK1, ERK

## Abstract

**Background:**

p21-activated kinase 1 (PAK1) plays a fundamental role in promoting the development and progression of several cancers and is a potential therapeutic target. However, the biological function and underlying mechanism of PAK1 in esophageal squamous cell carcinoma (ESCC) remain unclear.

**Methods:**

The expression of PAK1 was detected in both ESCC cell lines and clinical samples. Cell growth was measured by MTT, focus formation and soft agar assays. Cell migration and invasion were detected by wound healing and transwell assays. Animal models of subcutaneous tumourigenicity and tail vein metastasis were performed to determine the inhibitory effect of pharmacological inhibitor IPA-3 on tumor growth and metastasis of ESCC cells.

**Results:**

We found that PAK1 was frequently overexpressed in ESCC. Ectopic expression of PAK1 promoted cellular growth, colony formation and anchorage-independent growth. Overexpressing PAK1 also enhanced migration, invasion and the expression of MMP-2 and MMP-9 in ESCC cells. In contrast, silencing PAK1 by lentiviral knockdown or a specific inhibitor IPA-3 resulted in a contrary effect. Subsequent investigations revealed that Raf1/MEK1/ERK signaling pathway was involved in PAK1-mediated effect. Enhanced expression of Raf1 attenuated the inhibitory functions of PAK1 shRNA. Whereas blocking of Raf1 by shRNA or specific inhibition of MEK1 by U0126 antagonized the oncogenetic effect of PAK1 on ESCC cells. More importantly, Pharmacological inhibition of PAK1 by IPA-3 significantly suppressed tumor growth and lung metastasis of ESCC cells in vivo.

**Conclusions:**

These data support that PAK1 is an ideal target for the development of potential therapeutic drugs for ESCC patients even with metastasis.

**Electronic supplementary material:**

The online version of this article (10.1186/s12964-019-0343-5) contains supplementary material, which is available to authorized users.

## Background

Esophageal cancer is one of the most common gastrointestinal cancer and ranks as the sixth leading cause of cancer mortalities worldwide, with an estimated 456,000 new cases and 400,000 deaths in 2012 [1, 2]. According to their histological subtypes, the majority of esophageal carcinoma can be classified as esophageal adenocarcinoma (EAC) and esophageal squamous cell carcinoma (ESCC). Although enigmatic increases in the incidence of EAC have been observed among white populations in high-income countries, most patients in less-developed regions of the world especially in China are diagnosed as ESCC [3]. ESCC is associated with poor prognosis due to the limitation of clinical approaches for early diagnosis, high incidences of tumor recurrence and metastasis, as well as the ability of the tumor to acquire chemoresistance [4, 5]. Although 5-fluorouracil (5-FU) and cisplatin are consistently used as chemotherapy agents against ESCC at present, the 5-year overall survival rate is only approximately 25% [6]. Therefore, it is imperative to develop novel treatment regimens for this disease.

p21-activated kinase 1 (PAK1) is a serine/threonine protein kinase that serves as an important downstream mediator of the small Rho GTPases, including Rac1 and Cdc42, which regulates diverse cellular processes, including cell proliferation, anchorage-independent growth, cell adhesion, migration and invasion [7]. Amount evidence has indicated that PAK1 is substantially overexpressed in various cancers, including breast cancer, hepatocellular carcinoma, pancreatic cancer, lung cancer and cutaneous T cell lymphoma [8–11]. In addition, Ito et al reported that PAK1 mRNA expression is statistically associated with grade and the risk of recurrence in bladder cancers, and they also demonstrated that high PAK1 protein expression is an independent factor associated with recurrence [12]. Consistently, by using immunohistochemistry, Carter and colleges previously showed that PAK1 is increased with progression from the adenoma to carcinoma sequence, with the most obvious increases in invasive and metastatic human colorectal carcinomas [13]. More recently, Yang et al reported that overexpression of PAK1 correlates with aberrant expression of EMT markers and poor prognosis in non-small cell lung cancer [14]. All these findings indicate that PAK1 may play a central role in regulating tumorigenesis and metastasis. Indeed, ectopic expression of PAK1 facilitates the migration and invasion of gastric cancer cells. A recent study stated that PAK1 promotes epithelial–mesenchymal transition (EMT) and radio-resistance in lung cancer cells [15]. Conversely, knockdown of PAK1 by pharmacological inhibition and using short-hairpin RNA (shRNA) can significantly in hibit human cancer cell proliferation, anchorage-independent growth, migration and invasion in vitro and reduce tumor growth and metastasis in animal models [10, 11, 16–18]. In addition, depletion of active PAK1 up-regulates the immune system of APC^∆14/+^ mice and suppresses intestinal tumor development [19]. However, whether PAK1 is involved in ESCC development, progression and the underpinning molecular mechanisms remain unclear.

IPA-3 (2, 29-dihydroxy-1, 19-dinaphthyldisuifide), a highly selective, non-ATP-competitive allosteric inhibitor of PAK1, has been demonstrated to specifically prevent Cdc42-induced PAK1 autophosphorylation at threonine-423 (T423) and significantly inhibit PAK1 kinase activity [20]. A few studies showed that IPA-3 specifically blocks the membrane transport of WAVE2 and lamellipodia formation in human breast cancer cells [21], and inhibits the endocytic uptake of human adenovirus serotype 35 in various cell lines [22]. Wang et al found that inhibition of PAK1 by IPA-3 suppresses cutaneous T cell lymphoma (CTCL) cell proliferation and promotes spontaneous apoptosis, interestingly, the inhibitory effects of IPA-3 on CTCL cells is similar to that of the lentiviral-based PAK1 gene knockdown vectors [10]. Wong et al found that IPA-3 not only inhibits hepatocellular carcinoma (HCC) cell proliferation, colony formation and migration in vitro, but also suppresses tumorigenesis in a nude mouse xenograft model [23]. Furthermore, IPA-3 treatment also inhibits the growth of prostate xenografts in vivo [24]. More importantly, Selective inhibition of PAK1 using IPA-3 significantly abolishes TGFβ1-induced prostate cancer cell EMT and expression of mesenchymal markers [25]. However, the effect of IPA-3 in the therapeutic treatment of human ESCC is still poorly understood.

In the current study, we assessed the importance of PAK1 in ESCC. We found that inhibition of PAK1 by using shRNA and a small-molecule inhibitor (IPA-3) remarkably suppressed ESCC cell proliferation, colony formation, migration and invasion in vitro, and tumor growth and lung metastasis in vivo, at least in part, via down-regulating the Raf1/MEK1/ERK signaling pathway. Our studies highlight that PAK1 is a novel therapeutic target in ESCC and support the use of PAK1 inhibitors as a therapeutic strategy for this disease.

## Materials and methods

### Reagents

IPA-3 and U0126 were purchased from Selleck Chemicals (Shanghai, China). Antibodies and their sources are as follows: antibodies against p-PAK1 (T423, #2601), PAK1 (#2602), PAK2 (#4825), PAK3 (#2609), Rac1/Cdc42 (#4651), Raf1 (#9422), p-Raf1 (S338, #9427), MEK1 (#2352), p-MEK1 (S298, #9128), ERK1/2 (#4695), p-ERK1/2 (T202/Y204, #4370), MMP-2 (#4022) and MMP-9 (#3852) were obtained from Cell Signaling Technology (Beverly, MA). Primary antibodies against p-PAK2 (S141, #SAB4504634) and Actin (#4700) were purchased from Sigma-Aldrich (Shanghai, China).

### Cell culture

Human ESCC cell lines EC109, EC9706, KYSE30, KYSE70, KYSE150, KYSE450 and KYSE510 were obtained from the Cell Bank of the Chinese Academy of Sciences (Shanghai, China), and cultured in RPMI 1640 medium (Invitrogen) containing 10% (*v/v*) heat-inactivated fetal bovine serum (FBS) [26]. Het-1A (an immortalized human esophageal epithelial cell line), obtained from the American Tissue Culture Collection (Manassas, VA, USA), was cultured in DMEM culture medium supplemented with 10% FBS. All cells were cultured in a humidified incubator containing 5% CO_2_ and were recently tested for tested periodically for mycoplasma contamination and STR profiling.

### Human tissue specimens

The total 126 paraffin-embedded, archived samples (namely 63 pairs of ESCC and matched normal cases) used in this study were collected from the First Affiliated Hospital of Henan University between 2015 and 2018. All patients enrolled in the research had not received chemotherapy or radiation treatment. Patients’ age ranged from 50 to 85 years at the point of surgery. The research was approved by the Committees for Ethical Review of Research Involving Human Subjects in the First Affiliated Hospital of Henan University. Written informed consent was obtained from all patients prior to the study.

### RNA extraction and quantitative real-time PCR (qRT-PCR)

Total mRNAs were isolated using the TRIzol reagent (Invitrogen, Shanghai, China) following the manufacturer’s manual. Reverse transcription was carried out using PrimeScript RT Master Mix (TaKaRa, Dalian, China). qRT-PCR was performed using SYBR Premix Ex Taq II (TaKaRa, Dalian, China) on an ABI Prism 7, 900 System (Thermo Fisher Scientific). GAPDH served as internal control. The cycle threshold values did not differ by more than 0.5 among the triplicates. Relative expression differences were calculated using the 2^-ΔΔCt^ method. PCR primers sequences used are listed in Additional file 1: Table S1.

### Cell viability assay

Cell viability was determined by MTT assay. Briefly, cells were seeded in 96-well flat-bottom plates (Corning) and incubated with or without drugs for the indicated time. Twenty μL of MTT solution was added to each well during the last 4 h of culture. Absorbance was read on a 96-well plate reader at a wavelength of 490 nm. For ESCC cells treated with drugs, control cells received DMSO (< 0.1%) containing medium. The drug concentration resulting in 50% inhibition of cell growth (IC_50_) was determined by using GraphPad Prism version 7.0 (GraphPad Software, San Diego, CA).

### Focus formation assay

The colony formation assay was performed as previously described with some modification [27]. Briefly, a total of 500 cells each well were plated in six-well plates and cultured for 10–14 days (Ten days for EC109 and KYSE70; Two weeks for KYSE30 and KYSE150) until visible colonies formed. Surviving colonies were fixed with methanol and stained with 0.1% crystal violet in 20% methanol for 20 min. Microscopic colonies consisted of more than 50 cells were counted.

### Soft agar assay

Soft agar was carried out by growing 2, 000 cells in 0.4% bactoagar containing 10% FBS on a bottom layer of solidified 0.8% bactoagar (Sigma, Shanghai, China) with 10% FBS in a 24-well flat-bottom plate (Corning) as our previously described [26]. After incubation for 2–3 weeks at 37 °C in a humidified incubator, the colonies (containing ≥50 cells) were counted with an inverted optical microscope.

### Western blotting analysis

Western blotting analysis was performed using the whole cell lysates prepared in RIPA buffer (1 × PBS, 0.5% sodium deoxycholate, 0.1% sodium dodecyl sulfate, 1% NP-40). 10 mM β-glycerophosphate, 10 mM NaF, 1 × protease inhibitor cocktail (Roche, Indianapolis, IN) and 1 mM PMSF were added to the buffer mentioned above. Equal amounts of total proteins were separated using 10–15% SDS-PAGE, and then transferred onto nitrocellulose membranes. After blocking with 5% dried skimmed milk, the membranes were then incubated with the primary antibodies overnight. The appropriate HRP-linked secondary antibody was selected to detect the protein bands by using enhanced chemiluminescence (ECL) detection reagent (Beyotime, Shanghai, China).

### Wound healing assay

For the wound healing assay, cells were inoculated in a 6-well flat-bottom plate (Corning) and grown to a confluence cell monolayer for 12 h. After scratching with a pipette tip (200 μL), the wells were washed three times with sterile PBS to remove the floating cells. The cells were observed and captured at 0 h, 24 h and 48 h post-scratching. The percentage of wound closure was calculated by the formula: wound closure = (original gap distance - gap distance at the indicated time)/original gap distance × 100%.

### Cell migration and invasion assays

The migration and invasion assays were performed using transwell chambers (8 μm pore size, Costar) according to the manufacturer’s instructions as our previously described [26]. For migration assay, two hundred microliters of serum-free medium (containing 5 × 10^4^ cells) was placed into the upper chamber of each insert. Medium containing 20% FBS in the lower compartment served as the chemoattractant. After incubation at 37 °C for 24 h, the remaining tumor cells inside the upper chamber were removed with cotton swabs. The cells attached to the lower surface of the membrane insert were stained with 0.1% crystal violet after fixation with 4.0% paraformaldehyde, and then counted with an inverted microscope. The invasion assay was performed using a similar procedure, except that the upper surface of membrane inserts were coated with Matrigel (BD Biosciences, CA) to form a matrix barrier, and then 1 × 10^5^ tumor cells were added to the upper chamber.

### Transfection of plasmids and short hairpin RNA

Plasmids including pCMV-Flag-His-puro-PAK1, pCMV-Flag-His-puro-Raf1 and pCMV-Flag-His-puro empty vector were obtained from Transheep (Shanghai, China). For ectopic expression of PAK1, cells were transfected with 2 μg human PAK1-encoding plasmid or empty vector (pCMV-Flag-His-puro) using the Lipofectamine 2000 reagent (Invitrogen, Thermo Fisher Scientific, Inc.) according to the manufacturer’s instruction. Forty eight hours latter, the cells were then treated with puromycin (1.5 μg/mL, Sigma-Aldrich). After 4-week of selection, individual colonies were picked up and expanded. In order to stably overexpress Raf1, the PAK1-silenced KYSE30 and KYSE150 cells were transfected with 2 μg Raf1 and empty vector plasmid using the Lipofectamine 2000 reagent for 48 h. The cells were then exposed to puromycin (1.5 μg/mL) for 2 weeks.

Specific shRNAs targeting PAK1 or Raf1 and a scramble shRNA (pLKO.1-puro-Non-target shRNA) were purchased from Sigma-Aldrich. The indicated sequences were described in Additional file 2: Table S2. Lentiviruses production and subsequently tranfection we used were performed as previously described [26]. The cells were then selected in the presence of 1.5 μg/mL puromycin (Sigma-Aldrich) for 2 weeks to establish stable cells.

The efficiency of knockdown and overexpression were examined by immunoblotting.

### Mouse models of tumorigenesis and metastasis

Male nude BALB/c mice (18–20 g, 5 to 6 week-old) were purchased from Beijing Vital River Laboratory Animal Technology Co (Beijing, China). All mice were bred and maintained in barrier system at the animal facility of Henan University with controlled temperature (20 ± 2 °C), humidity (40–50%) and a lighting cycle of 12 h light/12 h darkness. Mice were housed in isolator cages (4 mice per cage). The standard pellet food and water were provided ad libitum during the experimental period. All the animal studies were approved by the Henan University Institutional Animal Care and Use Committee.

For in vivo tumorigenic experiment, KYSE150 cells (5 × 10^6^ cells in PBS suspension) were subcutaneously implanted into the left dorsal flank of each mouse [28]. Tumors were measured with calipers every other day and calculated by the following formula: tumor volume = *L* × *W*^2^ × 0.4, where *L* represents the smallest diameter and *W* is the diameter perpendicular to *L*. When tumors reached 100 mm^3^ in size, the mice were randomly split into control or experimental groups (*n* = 8 per group) and administrated by intraperitoneal injection (i.p.) with vehicle or IPA-3 (5 mg/kg), three times per week for 2 weeks. Mice were then anaesthetized with isoflurane before killed by cervical dislocation. Tumors were immediately removed, weighed, fixed and kept at − 80 °C. The body weight, feeding behavior and motor activity of each animal were monitored as indicators of general health.

For the experimental metastasis assay, KYSE150 cells (1 × 10^6^ in 100 μl PBS) were intravenously injected with via lateral tail vein into the tested nude mice (8 mice per group) as previously described [26]. After injection for 6 weeks, all of the mice were anaesthetized with isoflurane before being killed by cervical dislocation, the lungs were then harvested and fixed for 24 h in Bouin’s solution (5.0% acetic acid, 25% formaldehyde and 75% saturated picric acid). The metastatic colonies on the surface of the lung in each mouse were counted, and the presence of tumor lesions within the lungs was confirmed by H&E staining.

### Immunohistochemistry (IHC) and hematoxylin and eosin (H&E) staining

The formaldehyde-fixed tissues were dehydrated and embedded in paraffin according to the routine method. The paraffin-embedded tissues were then cut into pieces (4 μm) and placed on polylysine-coated slides. For IHC staining, the procedure was done as previously described in our previous studies. Briefly, The tissue slides were deparaffinized and rehydrated. The tissue slides were then treated with 3% H_2_O_2_ in for 10 min to exhaust endogenous peroxidase activity, the antigens were retrieved for 15 min in 10 mM citrate (pH 6.0) using a microwave oven. After 20 min of preincubation in 5% bovine serum albumin to prevent nonspecific staining, the slides were incubated overnight using primary antibodies against Ki67 (1:100), anti-p-PAK1 (1:200) and p-ERK1/2 (1:100) in a humidified container at 4 °C. Staining results were visualized by sequential incubations of slides with an EnVision + System-HRP (DAB; DAKO) was used according to manufacturer’s manual, followed by hematoxylin counterstaining. For staining with H&E, the paraffin-embedded tissue sections were deparaffinized in xylene and rehydrated with graded ethanol. Subsequently, the slides were stained with H&E by using Hematoxylin and Eosin Staining Kit (Beyotime, Shanghai, China). Images were photographed using a phase contrast microscope (Leica, Germany).

For human samples, IHC scoring was performed using a modified Histo-score (H-score) by two independent pathologists as our previously described [27]. the H-score included a semiquantitative assessment of both the proportion of positively stained cells and the intensity of staining. The proportion of positively stained cells was scored as 0–100%. The intensity score was defined according to the following standard: 0, no staining; 1, weak staining; 2, moderate staining; 3, strong staining. The intensity and fraction scores were then multiplied to obtain H-score (0–3), which represented the level of PAK1.

### Statistical analysis

All experiments were performed at least 5 times, and results were expressed as mean ± SD. Statistical analyses were performed using GraphPad Prism version 7.0 (GraphPad Software, San Diego, CA). A two-tailed Student’s *t* test was performed to compare the differences between two groups. We compared multiples groups with a one-way ANOVA with Tukey’s post hoc test, the overall F test was significant (*P* < 0.05), and there was no significant variance in homogeneity. All statistical tests were two-sided, and the *P* value of less than 0.05 was considered statistically significant.

## Results

### Overexpression of PAK1 is frequently detected in ESCC

**Fig. 1:**
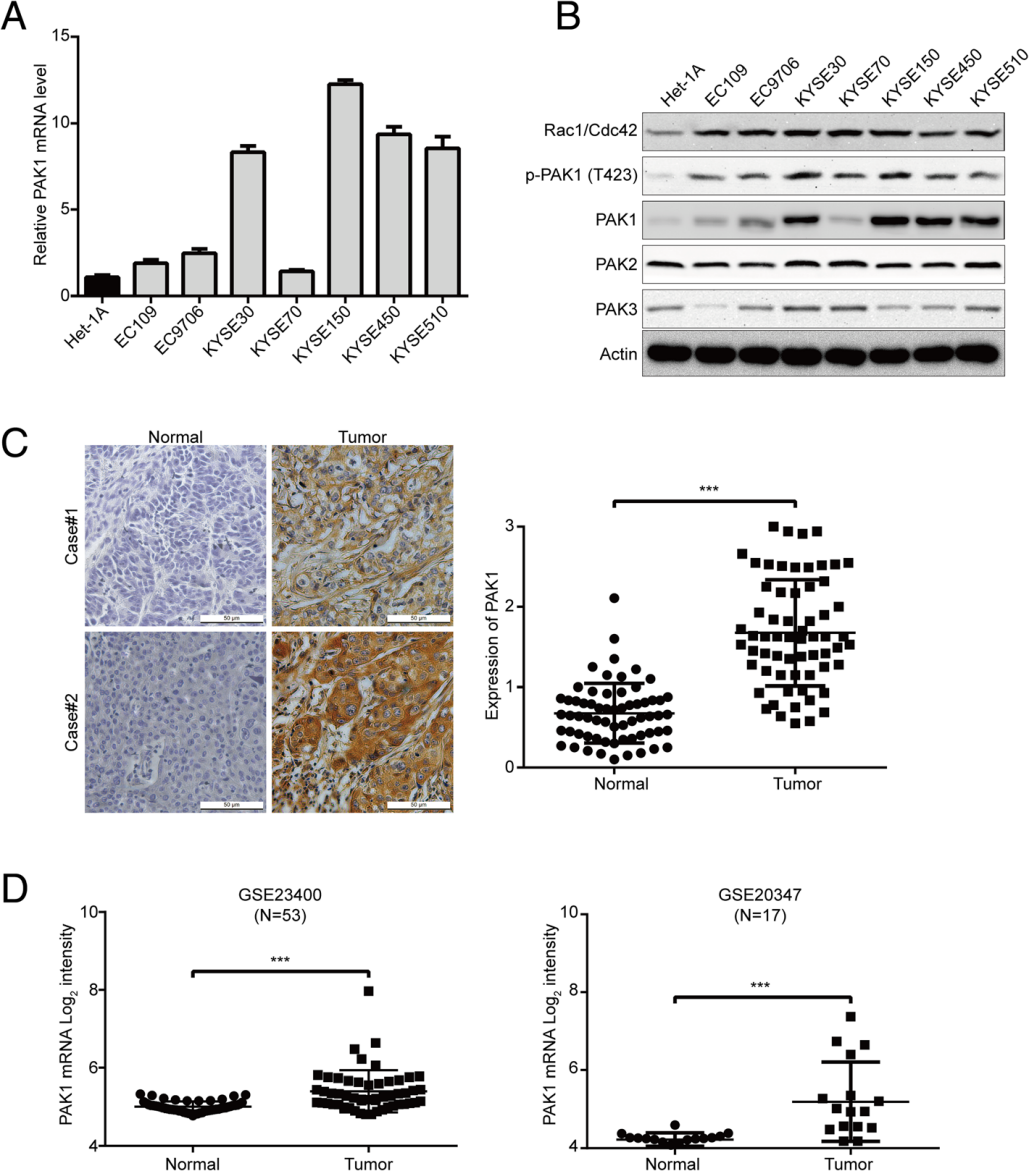
PAK1 is frequently overexpressed in ESCC. Expressions of PAK1 were detected by qRT-qPCR (**a**) and western blotting analysis (**b**) in one immortalized esophageal epithelial cell line (Het-1A) and seven ESCC cell lines. Data for qRT-qPCR represent the mean ± SD of six replicates. **c** Representative IHC micrographs (*left*) and summary bar chart (*right*) of PAK1 protein expression in ESCC tissues (all ESCC cases had matched adjacent normal tissues). Scale bars, 50 μm. **d** PAK1 mRNA expression was calculated from two data sets, GSE23400 (*N* = 53 pairs) and GSE20347 (*N* = 17 pairs), both of which were examined cDNA microarray from primary ESCC and paired normal tissues). ***P < 0.001 by paired Student’s t test

To determine the possible role of PAK1 in human ESCC, the levels of PAK1 mRNA in seven different ESCC cell lines were compared to that in one immortalized esophageal epithelial cell line (Het-1A) by using qPCR analysis. As shown in Fig. 1a, the mRNA expression of PAK1 were higher in six of seven ESCC cells (especially in KYSE30, KYSE150, KYSE450 and KYSE510 cells) compared with that of Het-1A cells. Western blotting results also showed that the protein levels of PAK1, p-PAK1 (T423), as well as its upstream mediators (Rac1 and Cdc42) were higher in ESCC cells than those in Het-1A cells. (Fig. 1b). To further confirm these findings, we detected the protein level of PAK1 by immunohistochemistry staining using 63 pairs of human ESCC and their adjacent normal specimens. As shown in Fig. 1c, PAK1 was dramatically upregulated in the ESCC tissues, but was only marginally detectable in normal esophageal tissues. Consistent with our results, the published microarray data (NCBI/GEO/GSE23400 and GSE20347) also showed that the mRNA expression of PAK1 was much higher in ESCC tissues compared with adjacent non-tumor tissues (Fig. 1d). These data suggests that PAK1 may be an oncogene in ESCC. Because lower expression level of PAK1 was observed in KYSE70 and EC109 cells, which were selected to use in PAK1-overexpressing experiments. KYSE30 and KYSE150 cells were used for PAK1 silencing study because their PAK1 expression level is relatively high.

### Knocking down PAK1 by shRNA inhibits ESCC cell growth

**Fig. 2:**
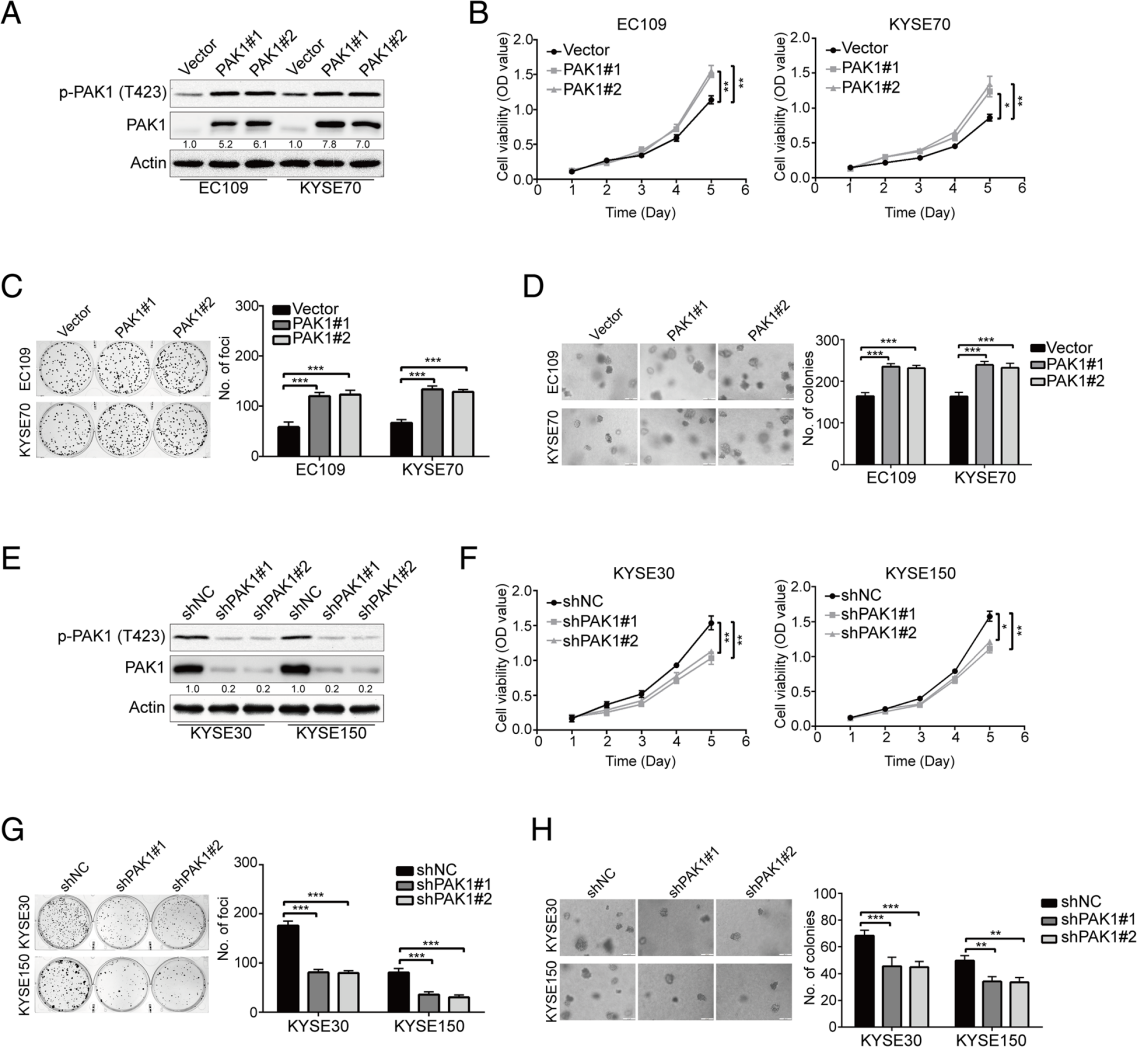
Targeting PAK1 by shRNA suppresses ESCC cell growth. **a** Western blotting analysis of PAK1 and p-PAK1 (T423) in single clones (PAK1#1 and PAK1#2) stably transfected with PAK1 in EC109 and KYSE70 cells. **b** Growth curve of indicated stable PAK1-overexpressing ESCC cells was detected by MTT cell proliferation assay (*n* = 8 per group). Representative images (*left*) and summary bar chart (*ritht*) of focus formation assay (*n* = 6 per group) (**c**) and colony formation in soft agar assay (n = 6 per group) (**d**) in ESCC cells stably transfected with PAK1 or empty vector (control). Scale bars, 200 μm. *P < 0.05, **P < 0.01 and ***P < 0.001 vs Vector group; *P* values were obtained by one-way ANOVA with post-hoc intergroup comparison with the Tukey’s test. **e** The effect of PAK1-targeting shRNAs was confirmed by Western blotting analysis. KYSE30 and KYSE150 were transfected with scrambled shRNA (shNC) or two shRNAs (shPAK1#1 and shPAK1#2) against PAK1. (**f**) The proliferation rate of the indicated stable PAK1-downregulated ESCC cells was examined by MTT assay (n = 8 per group). Silencing PAK1 could significantly decrease the frequency of focus formation (n = 6 per group) (**g**) and colony formation in soft agar (*n* = 6 per group) (**h**). Quantification (right panel) of colonies are depicted in the bar chart. Scale bars, 200 μm. *P < 0.05, **P < 0.01, ***P < 0.001 vs shNC; *P* values were obtained by one-way ANOVA with post-hoc intergroup comparison with the Tukey’s text

To explore the tumorigenic function of PAK1 in ESCC cells, we transfected EC109 and KYSE70 cells with PAK1 cDNA construct and collected single clones stably overexpressing PAK1 (PAK1#1 and PAK1#2). Empty vector-transfected cells (EC109-Vector and KYSE70-Vector were used as controls. Western blotting analysis showed that the expression of PAK1 and p-PAK1 (T423) was greatly upregulated in EC109 and KYSE70 cells that transfected with PAK1, compared with their control groups, respectively (Fig. 2a). The tumorigenic ability of PAK1 was first assessed by MTT cell growth assay. As illustrated in Fig. 2a, enforced expression of PAK1 elicited a left shift of the growth curve compared to their corresponding empty vector-transfected cells (Fig. 2b), indicating a higher cell viability. Focus formation assay also showed that colony formation frequency was significantly higher in PAK1-ovexpressing cells, compared to their corresponding control cells that transfected with empty vector (Fig. 2c). Further, we also performed colony formation assay in soft agar, which reflects an anchorage-independent growth in vitro. The results showed that colony formation frequency in soft agar was significantly increased in PAK1-expressing cells compared with their control groups (Fig. 2d).

We next determined whether knockdown of PAK1 inhibits the tumorigenicity in ESCC cells. To confirm this hypothesis, KYSE30 and KYSE150 cells were transduced with pLKO.1-shPAK1 lentivirus (shPAK1#1 and shPAK1#2, respectively). A scrambled shRNA (shNC) was used as a negative control. The silencing effect was checked by Western blotting analysis, and results showed that both shRNAs could specifically down-regulate the expression of PAK1 and p-PAK1 (T423) (Fig. 2e). Functional assays revealed that PAK1 silencing could effectively inhibit the tumorigenic phenotype by reducing cell growth, frequencies of focus formation and colony formation in soft agar compared with cells treated with shNC (Fig. 2f-h). Collectively, these results demonstrated that targeting PAK1 suppresses ESCC cell growth in vitro.

### PAK1 shRNA suppresses ESCC cell migration and invasion

**Fig. 3:**
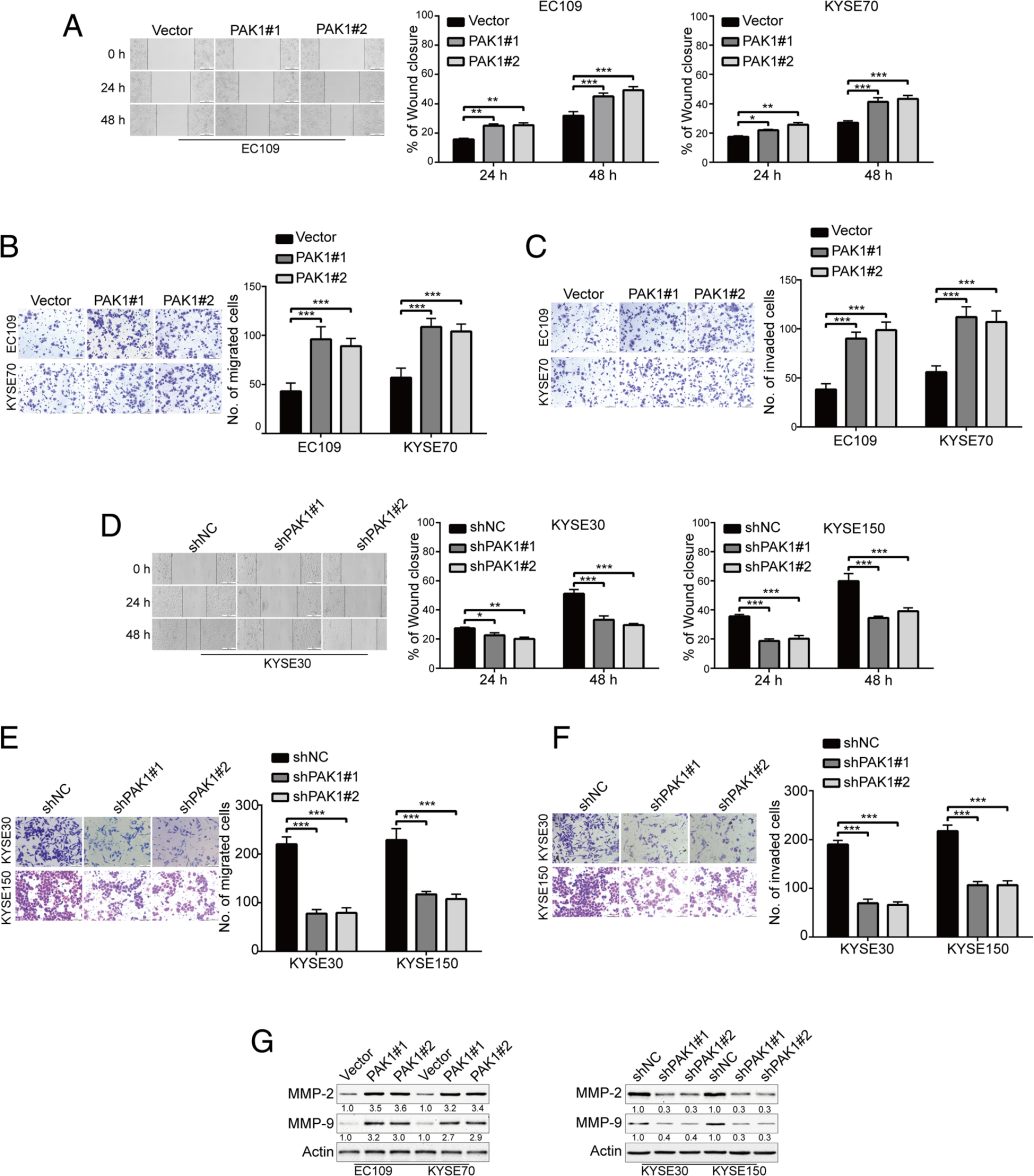
Silencing PAK1 decreases the migratory and invasive abilities of ESCC cells. **a** Overexpression of PAK1 dramatically promoted the migratory ability of ESCC cells in a wound-healing assay. Scale bars, 200 μm. Representative images (*left*) and summary bar chart (*right*) of cells that migrated through a membrane (**b**) or invaded through a Matrigel-coated membrane (**c**). Column and error bar represents mean ± SD (n = 6 per group). Scale bars, 100 μm. *, *P* < 0.05, **, *P* < 0.01 and ***, *P* < 0.001 vs Vector group; *P* values were obtained by one-way ANOVA with post-hoc intergroup comparison with the Tukey’s test. **d** PAK1 deletion dramatically reduced the migratory ability of ESCC cells in a wound-healing assay. Silencing PAK1 resulted in a decrease in the migratory (**e**) and invasive (**f**) abilities of ESCC cells as determined by Transwell analysis. Representative images (*left*) and summary bar chart (*right*) are shown. Column and error bar represents mean ± SD (n = 6 per group). *P < 0.05, **P < 0.01, ***P < 0.001 compared with shNC, one-way ANOVA, post-hoc intergroup comparisons, Tukey’s text. **g** Western blot analysis of MMP-2 and MMP-9 expression in PAK1-overexpresing and -silenced ESCC cells. Actin served as loading control

Metastasis is responsible for poor outcome of ESCC [29, 30]. We then investigated whether high expression of PAK1 confers ESCC cell abilities of migration and invasion, which are two of the most important processes in tumor metastasis. First, we examined the role of PAK1 in ESCC cell migration by using wound healing assay. As shown in Fig. 3a, enforced expression of PAK1 dramatically promoted the migration ability of EC109 and KYSE70 cells compared with those of their corresponding control cells at 24 h, the promoting effect of PAK1 was further evident at 48 h. To further identify these observations, we examined the effects of PAK1 on ESCC cell migration using the Boyden chamber transwell assay. Indeed, the migration ability of both lines of ESCC cells was significantly enhanced by PAK1 (Fig. 3b). Next, we determined whether high expression of PAK1 strengthens cell invasion by conducting transwell chamber with Matrigel. Our results showed that up-regulation of PAK1 significantly enhanced the number of EC109 and KYSE70 cells that invaded through Matrigel in comparison with those derived from their corresponding control cells transfected with empty vector (Fig. 3c). Interestingly, Western blotting analysis also displayed that overexpression of PAK1 remarkably promoted the expression of MMP-2 and MMP-9 (Fig. 3g, Left), which were well known to play a crucial role in tumor invasion and metastatic processes by degrading the extracellular matrix in various types of cancer including ESCC [31]. Above all, these results indicated that overexpression of PAK1 could significantly promote the migration and invasion of ESCC cells.

We next determined whether silencing PAK1 inhibits the migration and invasion of ESCC cells. Wound healing assay showed that downregulation of PAK1 significantly inhibited the migration ability of KYSE30 and KYSE150 cells compared with those of their corresponding control cells transfected with shNC at 24 h, the inhibitory effect of shPAK1 was further evident at 48 h (Fig. 3d). Moreover, the Boyden chamber transwell assay also showed that the migration ability of both lines of ESCC cells was significantly impaired by shPAK1 (Fig. 3e). Next, We performed transwell invasion assays to determine whether silencing PAK1 inhibits ESCC cell invasion. As illustrated in Fig. 3f, both ESCC cell lines that transfected with shPAK1 displayed a smaller number of tumor cells that invaded through Matrigel in comparison with those of their corresponding control cells transfected with shNC. In addition, Western blotting results showed that knockdown of PAK1 significantly reduced the expression of MMP-2 and MMP-9 (Fig. 3g, Right).

Taken together, these results demonstrated that targeting PAK1 by shRNA could suppress the migration, invasion, as well as the expression of MMP-2 and MMP-9 in ESCC cells.

### Raf1/MEK1/ERK signaling pathway plays an important role in PAK1-mediated ESCC cell growth, migration and invasion

**Fig. 4:**
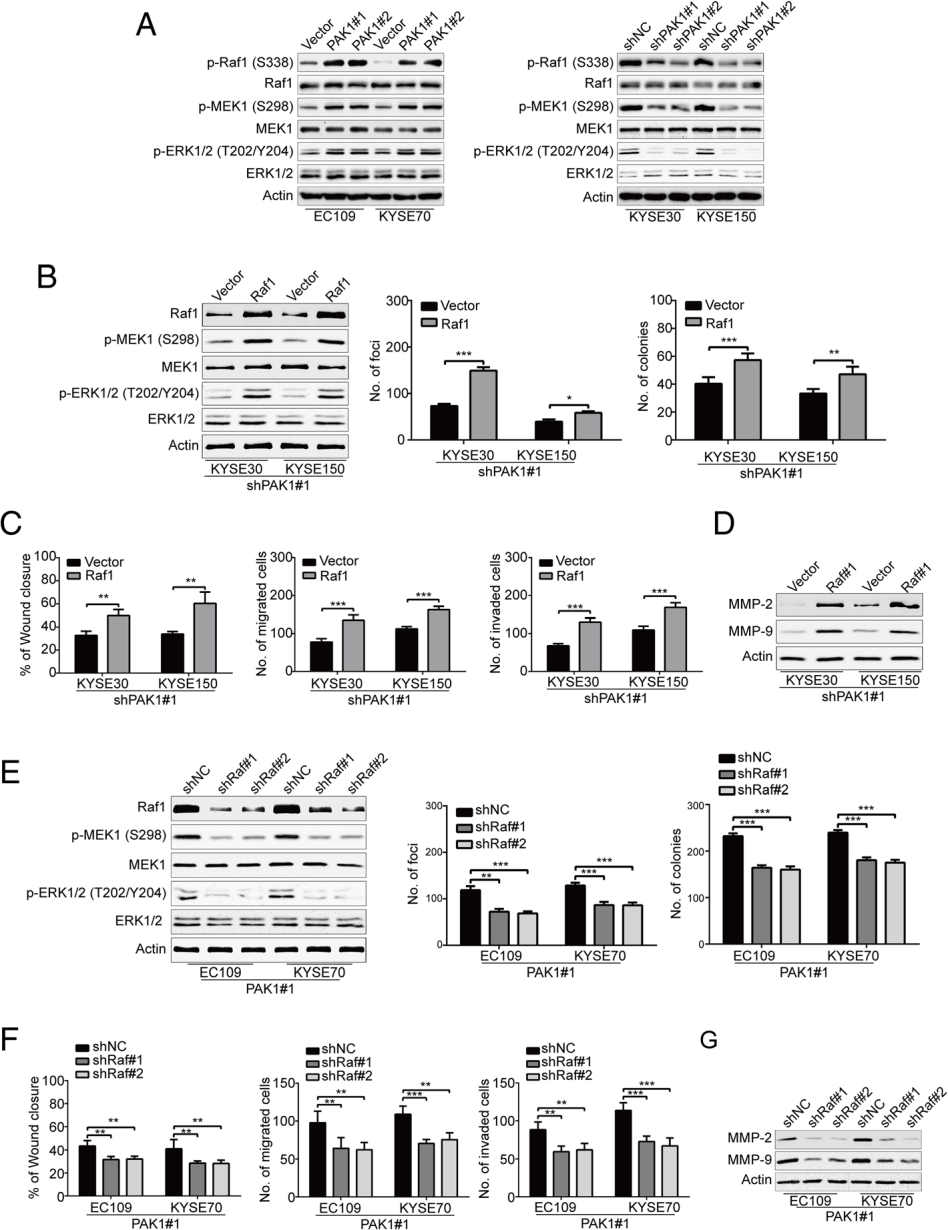
Raf1/MEK/ERK cascade is crucial for the aggressive cancer phenotype of PAK1 in ESCC cells. **a** Western blotting analysis was used to compare the protein levels of PAK1, Raf1, p-Raf1 (S338), MEK1, p-MEK1 (S298), ERK1/2, p-ERK1/2 (T202/Y204) in PAK1-overexpressing EC109 and KYSE70 cells, and in shPAK1-transfected KYSE30 and KYSE150 cells. Actin was used as loading control. **b** The effect of Raf1 shRNAs was confirmed by Western blot analysis (*left*). Silencing Raf1 significantly decreased the frequency of focus formation (*middle*) and colony formation in soft agar (*right*). Column and error bar represents mean ± SD (*n* = 6 per group). *P < 0.05, **P < 0.01, ***P < 0.001 by Student’s *t* test. **c** Raf1 knockdown dramatically reduced the migratory ability of PAK1-overexpressing EC109 and KYSE70 cells (*left*). Downregulation of Raf1 reduced the migratory (*middle*) and invasive abilities (*right*) of PAK1-overexpressing cells. Column and error bar represents mean ± SD (n = 6 per group). **P < 0.01, ***P < 0.001 by Student’s *t* test. **d** Knocking down of Raf1 attenuated MMP-2 and MMP-9 expression in PAK1-overexpressing EC109 and KYSE70 cells. **e** The efficiency of Raf1 overexpression in shPAK1-depleted KYSE30 and KYSE150 cells was confirmed by Western blot analysis (*left*). Enforced expression of Raf1 promoted the frequency of focus formation (*middle*) and colony formation in soft agar (*right*). Column and error bar represents mean ± SD (n = 6 per group). **P < 0.01, ***P < 0.001, one-way ANOVA with post-hoc intergroup comparison with the Tukey’s test. **f** Raf1 overexpression increased the migratory ability of shPAK1-depleted KYSE30 and KYSE150 cells (*left*). Raf1 overexpression enhanced the migratory (*middle*) and invasive abilities (*right*) of shPAK1-depleted KYSE30 and KYSE150 cells. Column and error bar represents mean ± SD (n = 6 per group). **P < 0.01, ***P < 0.001, one-way ANOVA with post-hoc intergroup comparison with the Tukey’s test. **g** Ectopic expression of Raf1 upregulated the protein levels of MMP-2 and MMP-9 in shPAK1-depleted KYSE30 and KYSE150 cells

We next explored the underlying mechanisms of how PAK1 influences the growth, migration and invasion of ESCC cells. It has been demonstrated that The Raf1/MEK1/ERK cascade plays a central role in ESCC biology [32–34], and PAK1 regulate ERK activity in some cell types by phosphorylating Raf1 at S338 and MEK1 at S298 [35–37], which suggests that the effect of PAK1 on ESCC cells involves the Raf1/MEK/ERK pathway. Intriguingly, Our results showed that ectopic expression of PAK1 in EC109 and KYSE70 cells increased the expression of p-Raf1 (S338), and its downstream targets: p-MEK1 (S298) and p-ERK1/2 (T202/Y204) (Fig. 4a, Left); In contrast, PAK1 knockdown in KYSE30 and KYSE150 cells significantly decreased the protein levels of p-Raf1 (S338), as well as p-MEK1 (S298) and p-ERK1/2 (T202/Y204) (Fig. 4a, Right).

We then asked whether Raf1/MEK1/ERK pathway participates in the effect of PAK1 in ESCC cells. First, we investigated whether Raf1 overexpression compensates for the loss of PAK1 function, a Raf1 cDNA expression construct was stably transfected into KYSE30-shPAK1#1 and KYSE150-shPAK1#1 cells (Fig. 4b, Left panel). Overexpression of Raf1 in PAK1-silenced KYSE30 and KYSE150 cells displayed higher focus formation frequencies and promoted colony forming abilities in soft agar compared with their corresponding control cells (Fig. 4b, Middle and Right panels). In addition, wound healing and Transwell migration and invasion assays also showed that ectopic expression of Raf1 significantly increased the migratory and invasive abilities in PAK1-knockdown KYSE30 and KYSE150 cells (Fig. 4c). Furthermore, Western blotting analysis demonstrated that enforced expression of Raf1 markedly rescued the expression of MMP-2 and MMP-9 in PAK1-silenced KYSE30 and KYSE150 cells (Fig. 4d).

Given that overexpression of Raf1 could rescue the loss of PAK1 function in ESCC cells, then we investigated the effect of Raf1 impairment on PAK1 function. In proliferation assays, the shRNA mediated downregulation of Raf1 in EC109-PAK1 and KYSE70-PAK1 cells showed lower focus formation frequencies and reduced colony forming abilities in soft agar (Fig. 4e). Consistently, wound healing and Transwell migration and invasion assays also showed that Raf1 knockdown resulted in a decrease in the migratory and invasive abilities in both PAK1-overexpressing ESCC cell lines (Fig. 4f). Western blotting analysis demonstrated that silencing Raf1 remarkably attenuated the expression of MMP-2 and MMP-9 in PAK1-overexpressing EC109 and KYSE70 cells (Fig. 4g).

**Fig. 5:**
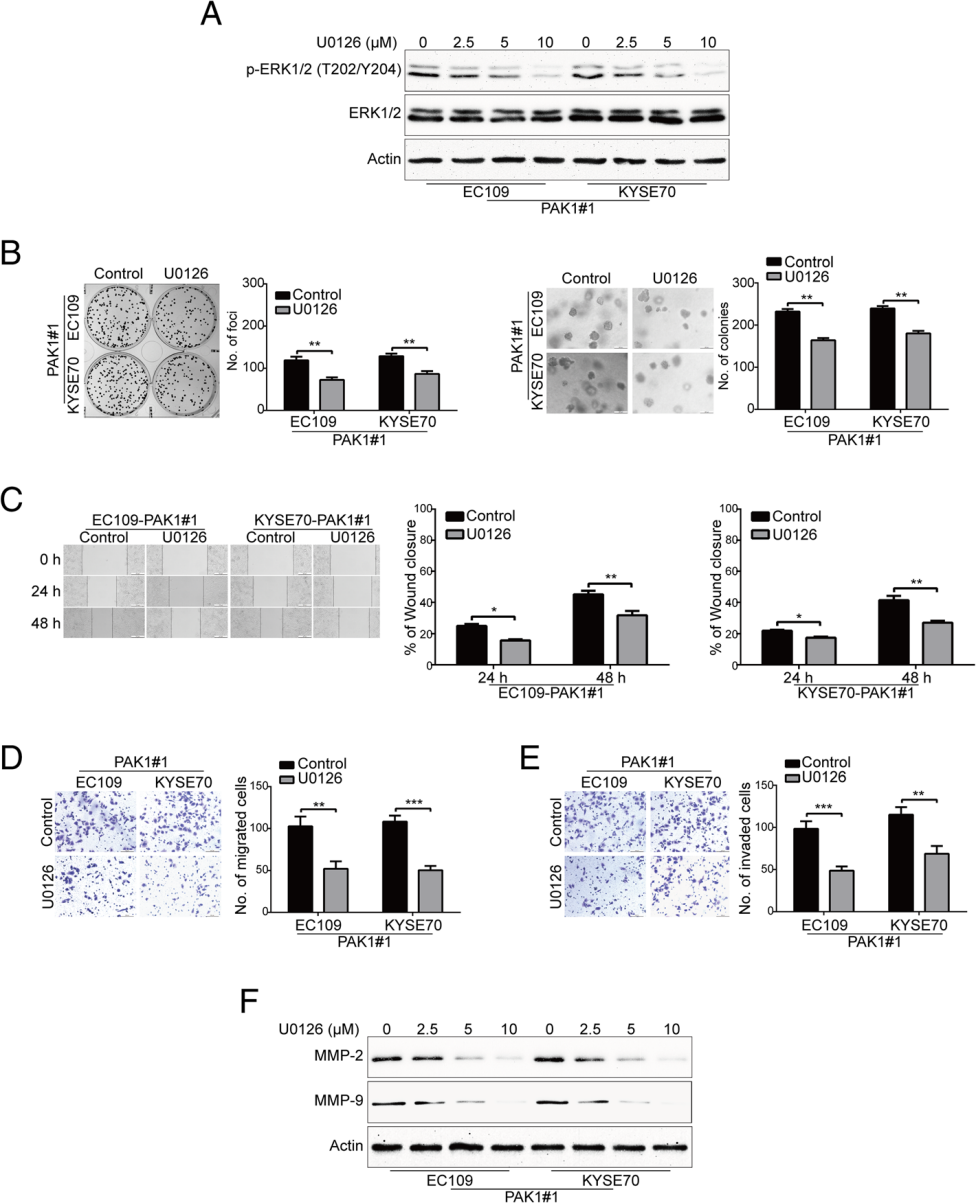
U0126 attenuates the aggressive effect of PAK1 on ESCC cells. **a** The effect of MEK inhibitor U0126 was confirmed by Western blot analysis. **b** U0126 significantly decreased the frequency of focus formation (*left*) and colony formation (*right*) in soft agar. PAK1-overexpressing KYSE30 and KYSE150 cells were exposed to 10 μM of U0126 for 48 h. The cells were then washed, and subjected to focus formation assay and soft agar assay. **c** U0126 dramatically reduced the migratory ability of PAK1-overexpressing EC109 and KYSE70 cells. PAK1-overexpressing KYSE30 and KYSE150 cells were exposed to 5 μM of U0126 for 0, and 48 h after scratching. The average gap width was used to evaluate migration. The wound breadth was normalized to the initial time point (0 h). Columns and error bars represent mean ± SD (n = 6 per group). *P < 0.05, **P < 0.01 by Student’s t test. Scale bars, 200 μm. **d** U0126 reduced the migratory and invasive abilities of PAK1-overexpressing EC109 and KYSE70 cells. PAK1-overexpressing KYSE30 and KYSE150 cells were treated with or without 5 μM U0126 for 48 h, and then underwent transwell migration and Matrigel invasion assays. Quantative analysis from 6 random fields. Scale bars: 100 μm. mean; error bar, SD. *P < 0.05; **P < 0.01 by Student’s *t* test. **e** U0126 diminished the protein levels of MMP-2 and MMP-9 in ESCC cells. The indicated PAK1-overexpressing cells were exposed to the various concentrations of U0126 for 48 h, the whole cell lysates were then subjected to Western blotting analysis to examine the expression of MMP-2 and MM-9

To further confirm that the oncogenic effect of PAK1 was through activation of the Raf1/MEK/ERK cascade, U0126, a selective MEK inhibitor, was applied to check whether it abolishes the effect of PAK1 in ESCC cells. Accordingly, results of Western blotting analysis showed that U0126 could effectively decrease expression levels of p-ERK1/2 (T202/Y204) (Fig. 5a). Interestingly, U0126 treatment resulted in lower colony formation frequencies and reduced colony forming abilities in soft agar in EC109-PAK1 and KYSE70-PAK1 cells (Fig. 5b). Moreover, wound healing assay showed that U0126 treatment attenuated the migratory abilities of PAK1-overexpressing ESCC cells (Fig. 5c). Furthermore, the migratory and invasive abilities of PAK1-overexpressing cells were significantly inhibited by U0126, compared with their corresponding control cells (Fig. 5d-e). Consistently, Western blotting analysis indicated that U0126 treatment dramatically inhibited the expression of MMP-2 and MMP-9 in a dose dependent manner in PAK1-overexpressing ESCC cells (Fig. 5f).

Taken together, these results further confirmed that Raf1/MEK1/ERK cascade is required for the effect of PAK1 in ESCC cells.

### Pharmacological inhibition of PAK1 inhibits ESCC cell growth, migration and invasion

**Fig. 6:**
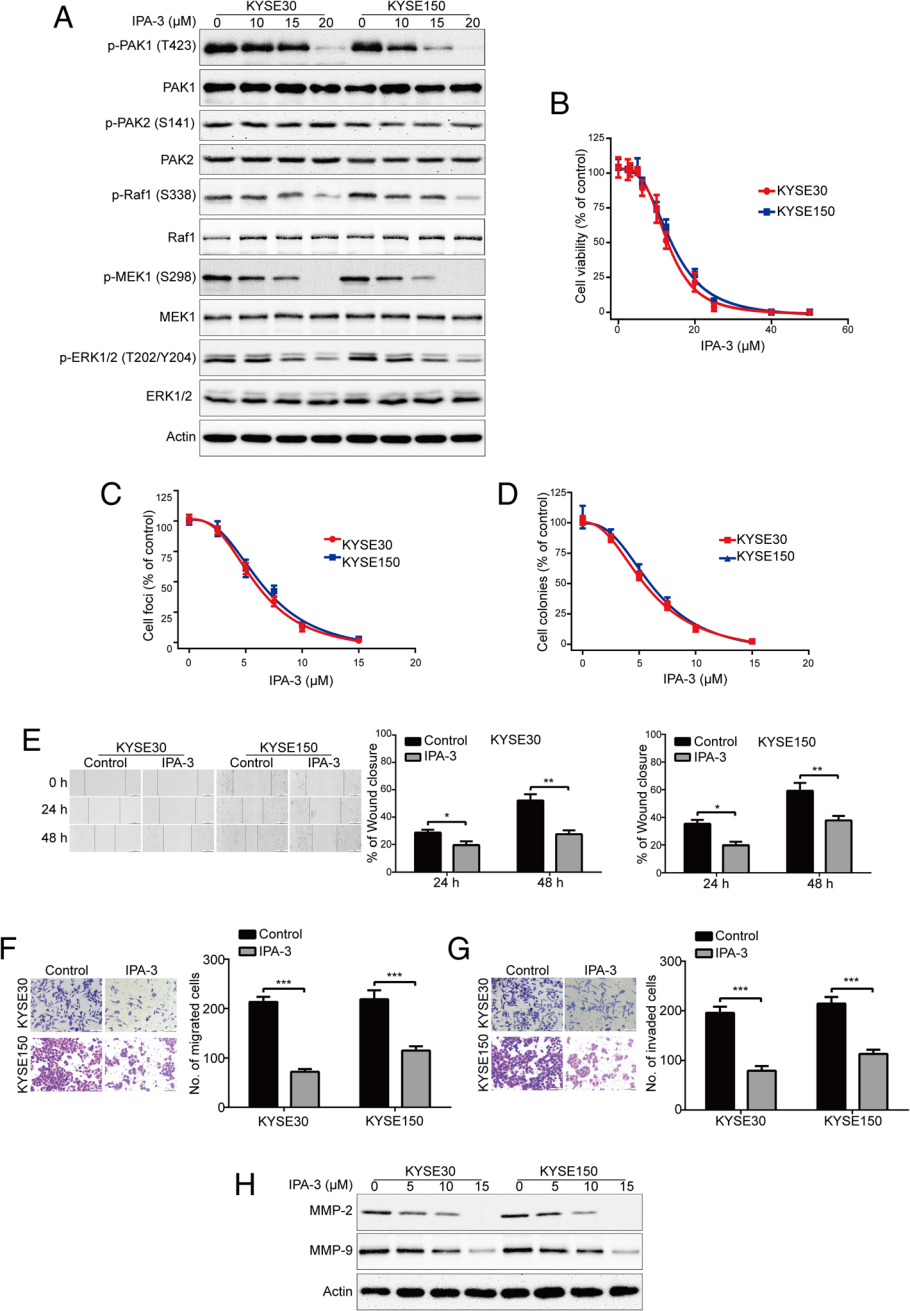
Pharmacological inhibition of PAK1 diminishes ESCC cell growth, migration and invasion. **a** KYSE30 and KYSE150 cells were treated with indicated concentration of IPA-3 for 48 h, and then the protein levels of p-PAK1 (T423), PAK1, p-PAK2 (S141), PAK2, Raf1, p-Raf1 (S338), MEK1, p-MEK1 (S298), ERK1/2, p-ERK1/2 (T202/Y204) were detected by Western blotting analysis. **b** The ESCC cells were exposed to increasing concentrations of IPA-3 for 72 h, the cell viability was measured by MTT assay. **c-d** KYSE30 and KYSE150 cells were exposed to increasing concentrations of IPA-3 for 48 h. The cells were then washed, and subjected to a drug-free focus formation assay (**c**) and soft agar assay (**d**). **e** KYSE30 and KYSE150 cells were treated with 10 μM of IPA-3 for 0, 24 h and 48 h after scratching. Scale bars: 200 μm. The average gap width was used to evaluate migration. Right, quantitative analysis of the wound relative breadth. The wound breadth was normalized to the initial time point (0 h). Columns and error bars represent mean ± SD (n = 6 per group). *P < 0.05, **P < 0.01, ***P < 0.001, one-way ANOVA with post-hoc intergroup comparison with the Tukey’s test. (F-G) KYSE30 and KYSE150 cells were treated with 10 μM IPA-3 for 48 h, and then underwent transwell migration (**f**) and Matrigel invasion (**g**) assays. *Left*, representative images; *Right*, quantative analysis from 6 random fields. Scale bars: 50 μm. mean; error bar, SD. ***P < 0.001 by Student’s *t* test. **h** IPA-3 diminished the protein levels of MMP-2 and MMP-9 in ESCC cells. KYSE30 and KYSE150 cells were exposed to the various concentrations of IPA-3 for 48 h, the whole cell lysates were subjected to Western blotting analysis

Since knockdown of PAK1 inhibits ESCC cell growth, colony formation, migration and invasion, we reasoned that PAK1 inhibitors may be effective as potential therapeutic agents for ESCC. IPA-3, an allosteric kinase inhibitor of PAK1 that covalently binds to the PAK1 regulatory domain and prevents the binding of the upstream activators (Rac1/Cdc42), thereby attenuating Cdc42-induced PAK1 autophosphorylation at threonine-423 (T423) and inhibiting PAK1 kinase activity [20]. As expect, IPA-3 treatment resulted in a significant reduction in the levels of phosphorylated PAK1 at T423, but not the phospho-PAK2 (S141) and the total amount of PAK1 and PAK2 (Fig. 6a). In addition, IPA-3 treatment also downregulated the expression of phospho-Raf1 (S338), phospho-MEK1 (S298) and phospho-ERK1/2 (T202/Y204) in a concentration dependent manner, while the total amount of Raf1, MEK1 and ERK1/2 proteins were not alternated (Fig. 6a). Next, we sought to determine whether pharmacological inhibition of PAK1 inhibits ESCC cell growth. MTT assay indicated that IPA-3 potently decreased the cell viability of KYSE30 and KYSE150 cells, with IC_50_ values of 12.81 μM and 13.94 μM, respectively (Fig. 6b). In addition, focus formation assay showed that IPA-3 potently inhibited the number of surviving clonogenic ESCC cells in a dose-dependent manner (Fig. 6c). Furthermore, soft agar assay also showed that the colony forming abilities in soft agar was significantly inhibited in KYSE30 and KYSE150 cells, with IC_50_ values of 5.81 μM and 6.35 μM, respectively (Fig. 6d). Overall, these data demonstrate that pharmacological inhibition of PAK1 by IPA-3 potently inhibits the growth of ESCC cells.

We next determined whether pharmacological inhibition of PAK1 by IPA-3 inhibits the migration and invasion in ESCC cells. Wound healing results indicated that the migratory abilities of KYSE30 and KYSE150 cells treated with IPA-3 were significantly inhibited at the dose of 10 μM for 24 h, the inhibitory effect of IPA-3 was further evident at 48 h (Fig. 6e). Moreover, Boyden chamber Transwell assay also showed that the migration ability of both ESCC cell lines was significantly impaired by IPA-3 at the concentration of 10 μM (Fig. 6f). We also analyzed whether IPA-3 inhibits cell invasion by conducting transwell invasion assays. As shown in Fig. 6g, IPA-3 strongly inhibited the invasion of KYSE30 and KYSE150 cells. Consistently, Western blotting analysis indicated that IPA-3 dramatically inhibited the protein level of MMP-2 and MMP-9 in a dose-dependent manner (Fig. 6h). These data indicated that targeting PAK1 by IPA-3 inhibits cell growth, as well as migration and invasion in ESCC cells.

### Targeting PAK1 by IPA-3 inhibits ESCC cell growth and metastasis

**Fig. 7:**
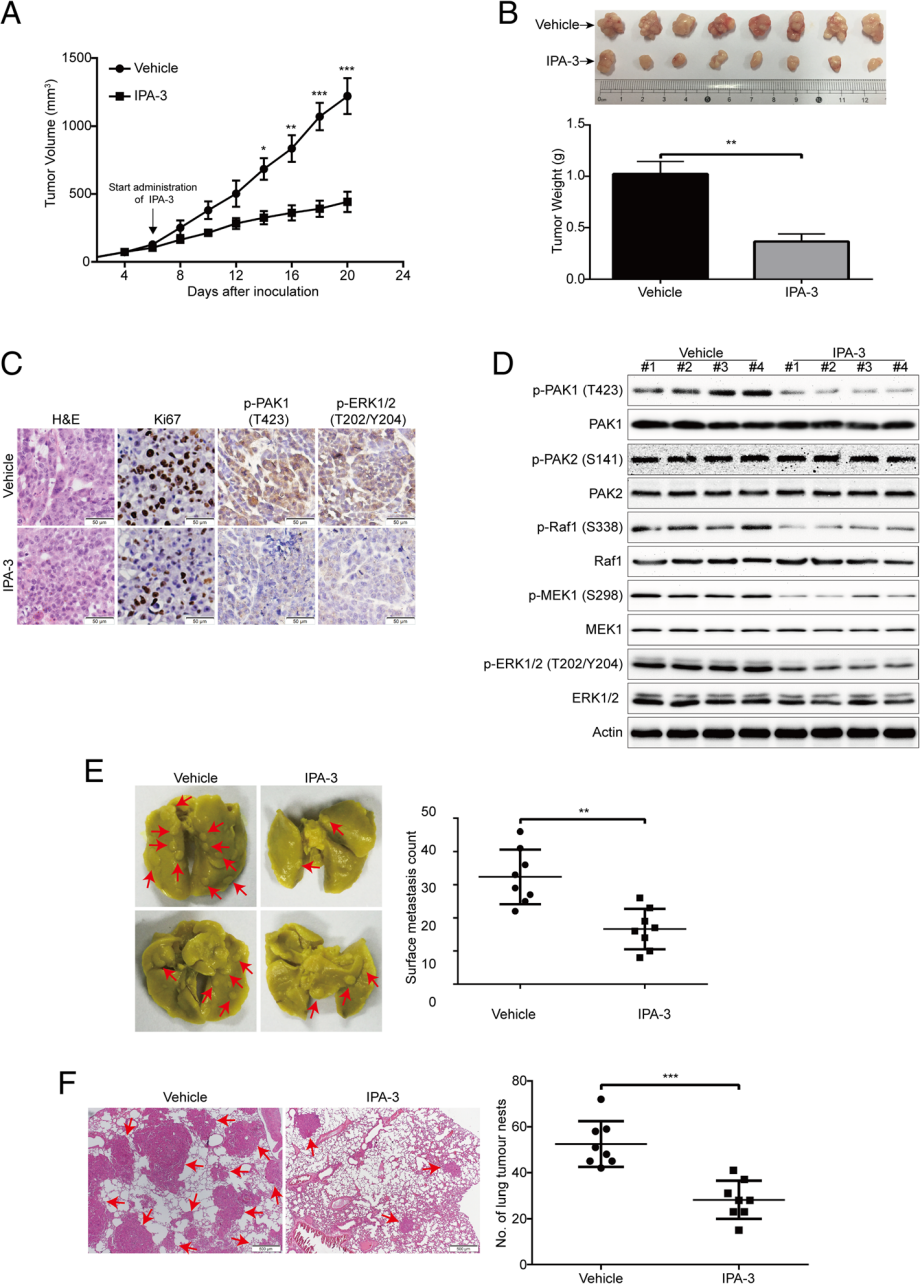
Targeting PAK1 by IPA-3 abrogates ESCC cell growth and metastasis in nude mice. **a** The growth curves of subcutaneous xenografts of KYSE150 cells are shown. Nude mice bearing KYSE150 xenograft tumors were treated with vehicle or IPA-3 (5 mg/kg) from day 6 to 20 after inoculation of KYSE150 cells. Points, mean; error bar, SD. *n* = 8 per group. *P < 0.05, **P < 0.01, ***P < 0.001 by Student’s *t* test. **b** After 14-day of IPA-3 treatment, the mice were sacrificed, tumors were dissected, weighed and photographed. Top, representative tumors from the control and experiment group are shown; bottom, comparison of tumor weights in control and experimental group (n = 8 per group). **P < 0.01 by Student’s *t* test. **c** Immunohistochemical analysis of p-PAK1 (T423), p-ERK1/2 (T202/Y204) and Ki67 in xenograft tissues from mice. Hematoxylin and eosin (H&E)-stained serial sections of the same xenografts are presented. Scale bars: 50 μm. **d** Immunoblotting of the p-PAK1 (T423), PAK1, p-PAK2 (S141), PAK2, Raf1, p-Raf1 (S338), MEK1, p-MEK1 (S298), ERK1/2 and p-ERK1/2 (T202/Y204) proteins in xenograft tissues is shown. **e** KYSE150 cells were intravenously injected into nude mice via the tail vein. Left, representative images of excised lungs after 6 weeks injection (arrows indicate the metastatic nodules). *Right*, graph showing the number of surface metastatic foci in the lungs. Error bars represent mean ± SD (n = 8 per group). **f** Lung metastases in each mice were confirmed by H&E staining. Arrows indicate the metastatic colonization of tumor cells in the lung tissues (*left panel*). The number of lung tumor nests in each group was counted and presented by mean ± SD (n = 8 per group) (*right panel*). Scale bars, 500 μm. **P < 0.01, ***P < 0.001 by Student’s *t* test

Given that targeting PAK1 by shRNA or pharmacology inhibitor IPA-3 could significantly suppress cell growth, migration and invasion in vitro, therefore, we want to determine whether targeting PAK1 suppresses ESCC cell growth and metastasis in vivo. For the subcutaneous xenograft experiment, KYSE150 cells were injected subcutaneously into the left dorsal flank of *nu/nu* BALB/c nude mice, six days later, when the tumor xenografts were palpable (about 100 mm^3^), the mice were randomly divided into two groups: vehicle or IPA-3 (8 mice per group) (5 mg/kg) for 2 weeks. The growth curve (tumor volume versus time curve) of KYSE150 tumors was significantly inhibited by the treatment of IPA-3 (Fig. 7a). In addition, the tumor weight in the IPA-3-treated mice was significantly lower than that of the vehicle-treated mice (Fig. 7b). In addition, assessment of the Ki67 proliferation marker by immunohistochemical staining showed that the proliferation of KYSE150 cells was significantly impaired by IPA-3. Similarly, the level of phospho-PAK1 (T423) and phospho-ERK1/2 (T202/Y204) was substantially diminished in the IPA-3-treated group compared with that from the vehicle-treated mice (Fig. 7c), which indicated that IPA-3 inhibited the kinase activity of PAK1. Consistent with these results, Western blotting analysis of cell lysates from xenografted tumors showed that IPA-3 dramatically inhibited the expression levels of phospho-PAK1 (T423), phospho-Raf1 (S338), phospho-MEK1 (S298) and phospho-ERK1/2 (T202/Y204), while the phospho-PAK2 (S141) and the total amount of PAK1, PAK2, Raf1, MEK1 and ERK1/2 proteins were not alternated (Fig. 7d).

Tumor metastasis is one of the important reasons that lead to poor outcome of patients with ESCC. Since targeting PAK1 by shRNA or pharmacology inhibitor IPA-3 drastically decreased the migration and invasive potential of ESCC cells in vitro, we next evaluated the effect of IPA-3 on metastasis of ESCC cells in vivo. Compared with vehicle treatment, IPA-3 significantly attenuated the ability of lung metastasis in nude mice, as evidenced by the smaller number of metastatic tumor nodules in the lungs (Fig. 7e). Furthermore, hematoxylin and eosin (H&E) results also showed an appreciable decrease in size and number of metastatic foci in the lungs of IPA-3-treated mice, compared with that of the vehicle-treated mice (Fig. 7f). These results indicated targeting PAK1 by IPA-3 significantly inhibited tumor metastasis in vivo.

Collectively, these findings indicate that targeting PAK1 by pharmacological inhibitor IPA-3 could suppress tumor growth and metastatic behavior of ESCC cells and that it likely acts through blocking the Raf1/MEK1/ERK signaling pathway.

## Discussion

In the present study, we found that PAK1 was frequently overexpressed in both ESCC cell lines and primary human ESCC tissues, suggesting that PAK1 may play an important oncogenic role in ESCC. Indeed, silencing PAK1 by shRNA or a specific inhibitor IPA-3 significantly suppresses ESCC cell proliferation, focus formation, anchorage-independent growth, migration, invasion and the expression of MMP-2 and MMP-9 in vitro. More importantly, Pharmacological inhibition of PAK1 by IPA-3 significantly suppressed tumor growth and lung metastasis of ESCC cells in vivo. Subsequent investigations revealed that Raf1/MEK/ERK signaling pathway was involved in PAK1-mediated effect. To our knowledge, this is the first report to elucidate the role of PAK1 in ESCC cells, using both in vitro and in vivo models.

P21-activated kinases (PAKs) are major effectors downstream of the small Rho family of GTPases. Among the six isoforms, PAK1 is the most ubiquitous and the best characterized member. Kim et al found that PAK1 is a candidate amplified loci in the xenograft established from the thoracic duct lymph of ESCC patients [38]. In addition, Chattopadhyay et al also found that PAK1 is one of the candidate genes located at amplified regions of chromosomes or low-level gain regions in patients with esophageal squamous cell carcinoma exposed to tobacco and betel quid from high-risk area in India [39]. However, the expression and biological function of PAK1 in ESCC remain unclear. In this study, by using multiple experimental methods, we found that PAK1 is strongly overexpressed in both ESCC cell lines and clinical samples at the mRNA and protein level. All these findings indicated that PAK1 performs as an oncogenic factor in ESCC. Indeed, both gain- and loss-of function studies demonstrated that high expression of PAK1 dramatically promotes ESCC cell growth, migration and invasion. Interestingly, we also found that pharmacological inhibition of PAK1 by IPA-3 significantly suppresses tumor growth and lung metastasis of ESCC cells in vivo and in vivo, suggesting PAK1 is promising therapeutic target for ESCC patients.

It is widely believed that Raf1/MEK1/ERK signaling pathway is one of the most commonly deregulated pathways in various cancer including ESCC. Several studies report that abnormal activation of the Raf1/MEK1/ERK signaling pathway plays an important role in promoting the growth, migration and invasion of ESCC cells [32–34]. PAK1 has been demonstrated to active Raf1/MEK1/ERK pathway in some cell types by phosphorylating Raf1 on serine 338 and MEK1 on serine 298 [36, 37, 40]. However, whether targeting PAK1 suppresses ESCC cell growth and lung metastasis via inhibiting the Raf1/MEK1/ERK pathway remain largely elusive. In this study, we found that ectopic expression of PAK1 in ESCC results in aberrant activation of Raf1/MEK1/ERK signaling pathway as monitored by p-Raf1 (S338), p-MEK1 (S298), p-ERK1/2 (T202/Y204). As expected, these proteins were down-regulated when PAK1 was silenced by shRNA or pharmacological inhibitor IPA-3. More importantly, we also found that enforced expression of Raf1 attenuates the inhibitory effect of IPA-3 on cell growth, colony formation, migration and invasion of ESCC cells. Conversely, silencing Raf1 by shRNAs or using U0126, a MEK1 specific inhibitor, dramatically abrogates the promotive effect of PAK1 on ESCC cells. Therefore, our study demonstrates that targeting PAK1 suppresses ESCC cell growth, migration and invasion at least partly via blocking Raf1/MEK1/ERK signaling pathway.

The poor prognosis of ESCC is mainly due to its high potential for metastasis, however, the underlying signaling pathways and key molecules that drive ESCC progress remain largely unknown. In this study, we found that ectopic expression of PAK1 significantly promotes the cell motility and invasion of ESCC cells, as well as the expression of MMP-2 and MMP-9. Consistent with our results, overexpression of PAK1 is associated with progression, metastasis and prognosis of gastric cancer [41]. Similarly, overexpression of PAK1 also correlates with aberrant expression of EMT markers and poor prognosis in non-small cell lung cancer [14]. In addition, PAK1 is highly expressed in a proportion of pancreatic adenocarcinoma patients and its expression is significantly associated with MET positivity and linked to a widespread metastatic pattern [18]. These data indicate that PAK1 plays an important role in promoting tumor metastasis, therefore targeting PAK1 may significantly suppresses the metastasis of these cancer. Indeed, knocking down of PAK1 significantly reduces cell adhesion, migration, and invasion of gastric cancer cells in vitro and significantly prevents tumor metastasis in vivo [37]. Moreover, Inhibition of PAK1 by shRNA or pharmacology inhibitor IPA-3 also attenuates cell migration in vitro and tumor metastasis in a model of pancreatic adenocarcinoma [18]. Targeting PAK1 could significantly suppress the metastasis of ESCC cells. This conclusion is based on the following evidence. First, Conversely, knockdown of PAK1 by shRNAs or pharmacological inhibitor IPA-3 leads to opponent trends. More importantly, pharmacological inhibition of PAK1 by IPA-3 could obviously suppress the lung metastasis of ESCC cells in a nude mouse xenografted model. Our data suggests that PAK1 is a promising therapeutic target for ESCC patients, even with metastasis.

It is well known that matrix metalloproteinases (MMPs) play an important role in the degradation of extracellular matrix (ECM) components, which facilitating the invasion and metastasis of tumor cells [42]. both MMP-2 and MMP-9, two important members of MMPs, have been extensively studied. Overexpression of these two factors is observed in malignant tumors and positively correlates with the aggressive malignant phenotypes and poor prognosis in ESCC patients [31, 43]. Goc et al also demonstrated that MMP-9 is involved in PAK1-mediated prostate tumor growth and microinvasion [44]. In addition, a recent study also indicated that PAK1 promotes the invasion of gastric cancer cells by inducing the mRNA expression and activity of MMP-2 [45]. Whether PAK1 can display a similar function in ESCC cells remain unclear. Interestingly, in this study, we found that ectopic expression of PAK1 significantly enhances the protein level of MMP-2 and MMP-9. And vice versa, both MMP-2 and MMP-9 expression is concomitantly downregulated upon PAK1 knockdown by shRNAs or inhibition by a specific inhibitor IPA-3. More importantly, we also found that overexpression of Raf1 attenuates the inhibitory effect of PAK1 inhibitor IPA-3 on MMP-2 and MMP-9 expression. Conversely, silencing Raf1 by shRNAs or using U0126, a MEK specific inhibitor, dramatically abrogates the promotive effect of PAK1 on the protein level of MMP-2 and MMP-9. Therefore, it is possible that PAK1 downregulation or inhibition leads to the inhibition of Raf1/MEK1/ERK signaling pathway, which in turn blocks the expression of MMP-2 and MMP-9. Our data are in line with a previous study showing that inhibiting ERK1/2 activity by U0126 significantly suppresses the transcription activity of MMP-2 by inhibiting the phosphorylation of hnRNPK and its nuclear translocation in colorectal cancer (CRC) cells [46]. Similarly, U0126 completely abolished globular domain isoform of CTRP9 (gCTRP9)-induced upregulation of MMP-9 and phosphorylated ERK1/2 in adiposederived mesenchymal stem cells [47]. However, Qin et al reported that treatment with MEK1/2 inhibitor U0126 in Peptidylargininedeiminase 1 (PAD1) knockdown cells significantly recovers MMP-2 expression, but has no effect on MMP-9 in human triple negative breast cancer (TNBC) cells [48]. Taken together, our results in combined with these findings from others, suggesting that the effect of Raf1/MEK1/ERK signaling pathway on the expression of MMP-2 and MMP-9 strongly depends on the cellular type context.

Previous reports showed that cancer cells that carry mutations in RAS and Rac1 genes are more sensitive to PAK inhibitors [49, 50]. In addition, several lines of evidences indicate that the KRAS mutation was detected in a few ESCC patients [51–53]. Although the activating mutations in RAC1 gene in ESCC has not been published to date, we and others had found Rac1 protein is consistently higher in ESCC cell lines and tissues compared with the corresponding control group [54]. Moreover, the high expression of Rac-1 is significantly correlated with advanced tumor depth, lymph node metastasis, and shorter disease-free and survival time [55]. In this study, we also demonstrated that targeting PAK1 by shRNA or pharmacology inhibitor IPA-3 could significantly suppress ESCC cell growth, migration, invasion and metastasis in vitro and in vivo. Therefore, targeting PAK1 may be a promising therapeutic strategy for ESCC patients, especially for those with RAS and Rac1 mutations.

## Conclusions

The present study has identified PAK1 is an oncogene in ESCC. Mechanistic analysis demonstrated that targeting PAK1 inhibits ESCC cell growth, colony formation, anchorage-independent growth, migration, invasion and metastasis, at least in part through blocking the Raf1/MEK1/ERK signaling pathway. These findings provide evidence for the first time that overexpressed PAK1 is an important molecule participating in the aberrant activation of Raf1/MEK1/ERK signaling pathway and thus may serve as a therapeutic target for ESCC. Our results warrant a clinical trial to further evaluate the efficacy of PAK1 inhibitors in ESCC patients even with metastasis.

## Additional files

Additional file 1: **Table S1.** Primers used for qRT-PCR. (PDF 85 kb)

Additional file 2: **Table S2.** Primers used for shRNA. (PDF 42 kb)

## Abbreviations

5-FU: 5-fluorouracil
EAC: Esophageal adenocarcinoma
EMT: Epithelial–mesenchymal transition
ERK: Extracellular regulated protein kinases
ESCC: Esophageal squamous cell carcinoma
H&E: Hematoxylin and eosin
IHC: Immunohistochemistry
MMP-2: Matrix metalloproteinase 2
MMP-9: Matrix metalloproteinase 9
PAK1: p21-activated kinase 1
qRT-PCR: Quantitative real-time PCR
shRNA: Short-hairpin RNA
