# Supplementary material for: Targeting p21-activated kinase 1 inhibits growth and metastasis via Raf1/MEK1/ERK signaling in esophageal squamous cell carcinoma cells
Source: Cell Commun Signal. 2019 Apr 11;17:31. doi: 10.1186/s12964-019-0343-5 (PMC6458688; doi:10.1186/s12964-019-0343-5)
Supplement: Supplementary file 1 — Table S1. Primers used for qRT-PCR. (PDF 85 kb) [file 12964_2019_343_MOESM1_ESM.pdf]

Supplementary Table S1.

Table S1. Primers used for qRT-PCR.

Gene	Forward	Reverse
**PAK1**	5’-CGCAGGCTGTTCTGGATGT-3’	5’-GTGGCACTGCAGGAGTCTCA-3’
**GAPDH**	5'- GAAGGTGAAGGTCGGAGTC-3'	5'- GAAGATGGTGATGGGATTTC-3'
