# Supplementary material for: Targeting p21-activated kinase 1 inhibits growth and metastasis via Raf1/MEK1/ERK signaling in esophageal squamous cell carcinoma cells
Source: Cell Commun Signal. 2019 Apr 11;17:31. doi: 10.1186/s12964-019-0343-5 (PMC6458688; doi:10.1186/s12964-019-0343-5)
Supplement: Supplementary file 2 — Table S2. Primers used for shRNA. (PDF 42 kb) [file 12964_2019_343_MOESM2_ESM.pdf]

Supplementary Table S2.

Table S2. Primers used for shRNA.

Gene	Sequence (5' to 3')
**shNC (No target shRNA)**	CCGGGCGCGATAGCGCTAATAATTTCTCGAGAAATTATTAGCGCTATCGCGCTTTTT
**shPAK1#1**	CCGGGCATTCGAACCAGGTCATTCACTCGAGTGAATGACCTGGTTCGAATGCTTTTTTG
**shPAK1#2**	CCGGCCCTAAACCATGGTTCTAAACCTCGAGGTTTAGAACCATGGTTTAGGGTTTTTTG
**shRaf1#1**	CCGGCATGAGTATTTAGAGGAAGTACTCGAGTACTTCCTCTAAATACTCATGTTTTT
**shRaf1#2**	CCGGGCTTCCTTATTCTCACATCAACTCGAGTTGATGTGAGAATAAGGAAGCTTTTT
